# “Penumbra sign” in knee pain: a case of distal femur osteomyelitis

**DOI:** 10.1590/1414-431X2023e12976

**Published:** 2024-02-09

**Authors:** Hakan Koray Tosyali, Adem Çakir

**Affiliations:** 1 Department of Orthopedics & Traumatology, Celal Bayar University, Medical Faculty, Manisa, Turkey; 2 Department of Emergency Medicine, Çanakkale Mehmet Akif Ersoy State Hospital, Çanakkale, Turkey

**Keywords:** Penumbra sign, Osteomyelitis, Bone tumors, Pain, Emergency medicine

## Abstract

“Penumbra sign” is a characteristic finding in magnetic resonance imaging (MRI) of Brodie's abscess, a rare variant of subacute osteomyelitis. We aimed to discuss the imaging finding penumbra sign that will help in the diagnosis of osteomyelitis and may be useful to clinicians in differential diagnosis. A 26-year-old male patient presented to the emergency department with complaints of pain and limping in the right knee that did not go away. He had a history of arthroscopic debridement and percutaneous fixation surgery due to osteochondral fragment 3 years ago. There were no additional findings in the patient's vital parameters, physical examination, and medical history. X-ray imaging revealed two screws in the distal femur and a well-defined sclerotic rim surrounding a radiolucent lesion anterior to the screws. MRI revealed a lesion in the distal femoral metaphysis with low-density fluid and hyperintense granulation tissue surrounding it. After surgical abscess drainage and local debridement, bone cement was placed in the resulting cavity. Teicoplanin treatment was started. The patient was discharged and complete recovery was achieved in the second month. The diagnosis of osteomyelitis is often missed or confused with bone tumors in non-traumatic cases presenting with persistent bone pain. MRI imaging is frequently used in differential diagnosis, and detection of characteristic imaging signs such as the penumbra sign accelerates the diagnosis. In this context, emergency department clinicians, in particular, should be cautious and not forget that early treatment can be started by recognizing these signs.

## Introduction

Brodie's abscess is a term coined after a case of tibia abscess that resulted in the amputation of the limb of the patient who had no systemic disease or symptoms. Sir Benjamin Brodie first used this term to describe a localized bone abscess that develops without any systemic disease (1). Brodie's abscess is a rare variant of subacute osteomyelitis that is atypical and has late onset. It may have an insidious onset due to the lack of characteristic symptoms or due to mild symptoms (2-4). Symptoms can be diverse, including localized pain especially at night, mild functional impairment, and localized swelling, redness, and tenderness (5). To make a diagnosis, clinicians must first have a suspicion of this disease. Imaging methods are required to confirm the diagnosis, ranging from radiography to magnetic resonance imaging (MRI).

Roberts et al. (6) classified osteomyelitis into six forms. Brodie's abscess, which has a characteristic penumbra sign, is classified as type Ib and is a cavity filled with pus or granulation tissue, surrounded by a dense fibrous tissue layer and a sclerotic area of the metaphysis bone. This appearance often leads to misdiagnosis of various benign and malignant bone tumors (7).

Brodie's abscess is commonly seen in young men. It is most frequently seen in the tibia and distal femur, especially in the second or third decade of life (3,8). Rare cases have been reported in the talus, proximal femur, cuboid bones, fibula, clavicle, and pelvis (8-12).

In this case report, we aim to discuss a Brodie's abscess in a 26-year-old male patient, and the penumbra sign image that can be helpful in diagnosing this pathology and provide differential diagnosis for clinicians.

## Case presentation

### Case

The patient was a 26-year-old male who presented to the emergency department with complaints of pain in his right knee. After evaluation, no fracture or dislocation was observed, so he was treated with analgesic/anti-inflammatory therapy and discharged.

**Figure 1:**
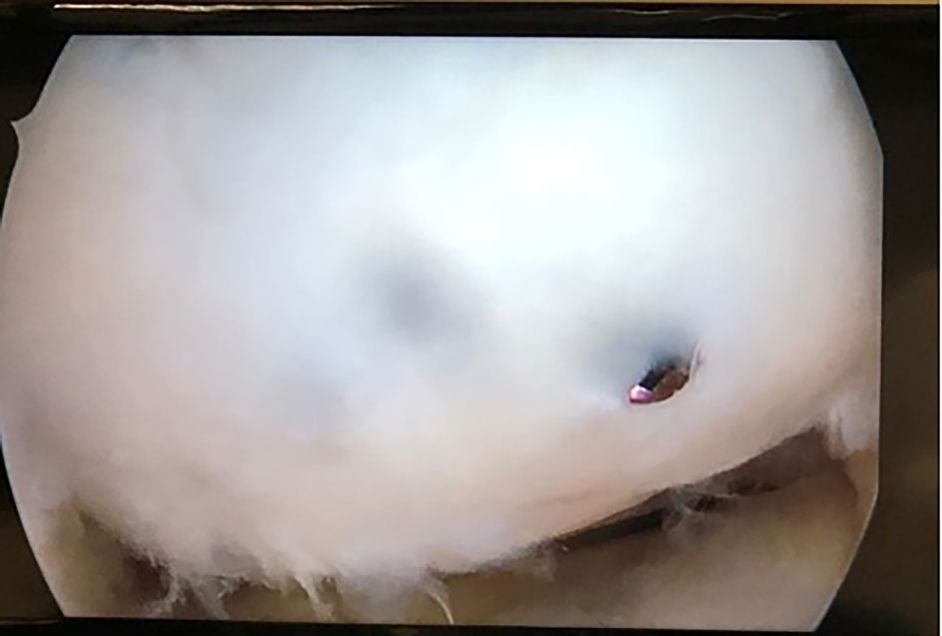
Cystic lesion observed during the patient's arthroscopic debridement procedure.

**Figure 2:**
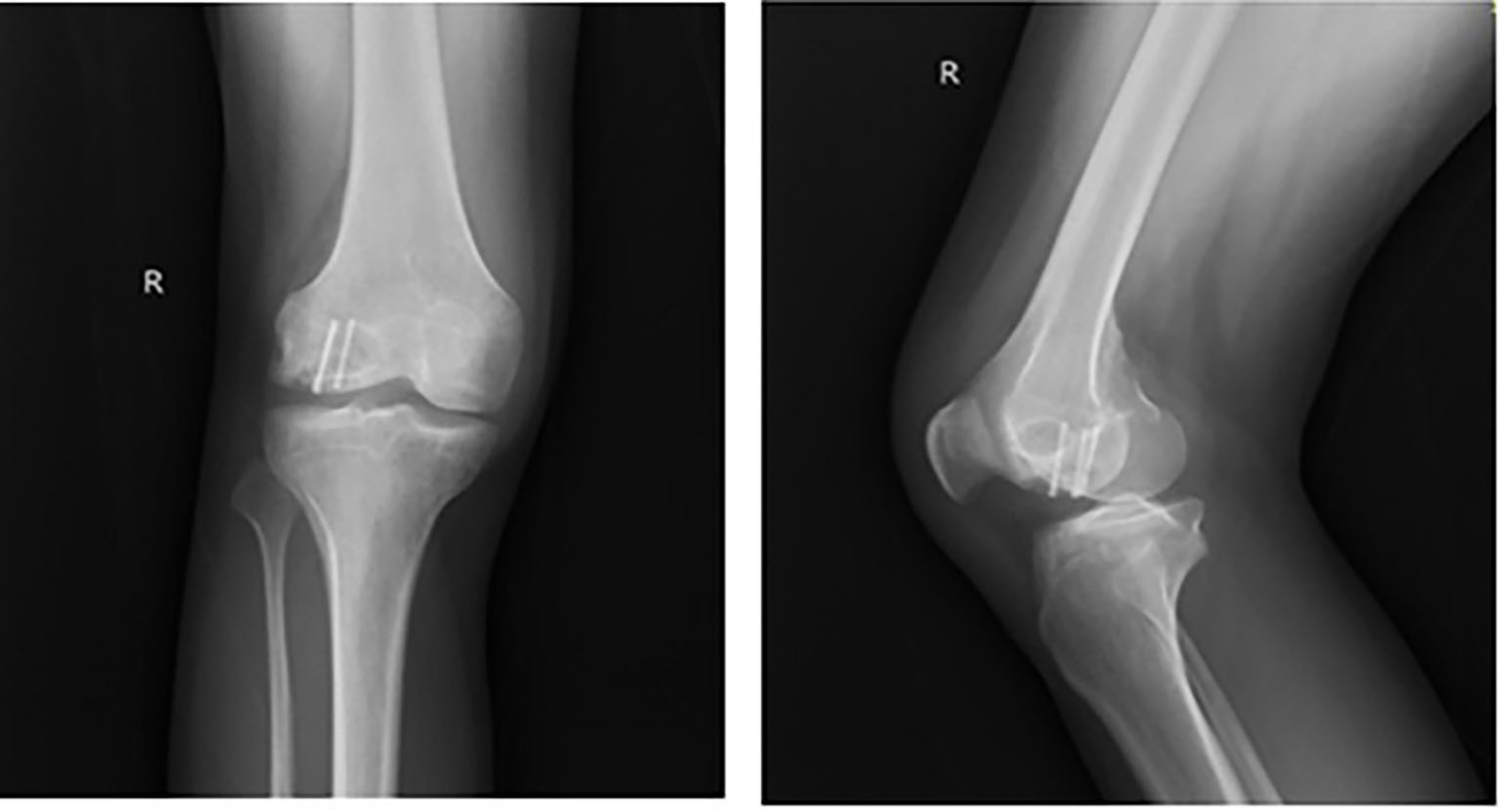
Pinning procedure seen on the X-ray imaging of the patient.

One week later, he returned to the emergency department with continued pain in his right knee. Due to symptom recurrence and lack of improvement, a detailed evaluation was performed. His history revealed that he had been experiencing pain in his right knee for three years following a sports injury, and after evaluation by an orthopedic specialist, an osteochondral fragment was suspected. Medical treatment was unsuccessful, and surgery was planned. The review of the patient’s medical records showed that he underwent arthroscopic debridement and percutaneous fixation (Acutrak headless compression screw) (Figure 1). Imaging tests also showed the fixation procedure performed on the patient (Figure 2).

After the procedure, no complications were observed during follow-up, and the patient was discharged with a good prognosis and scheduled for further check-ups. During follow-up visits, he reported doing well, and evaluation was within normal limits.

**Figure 3:**
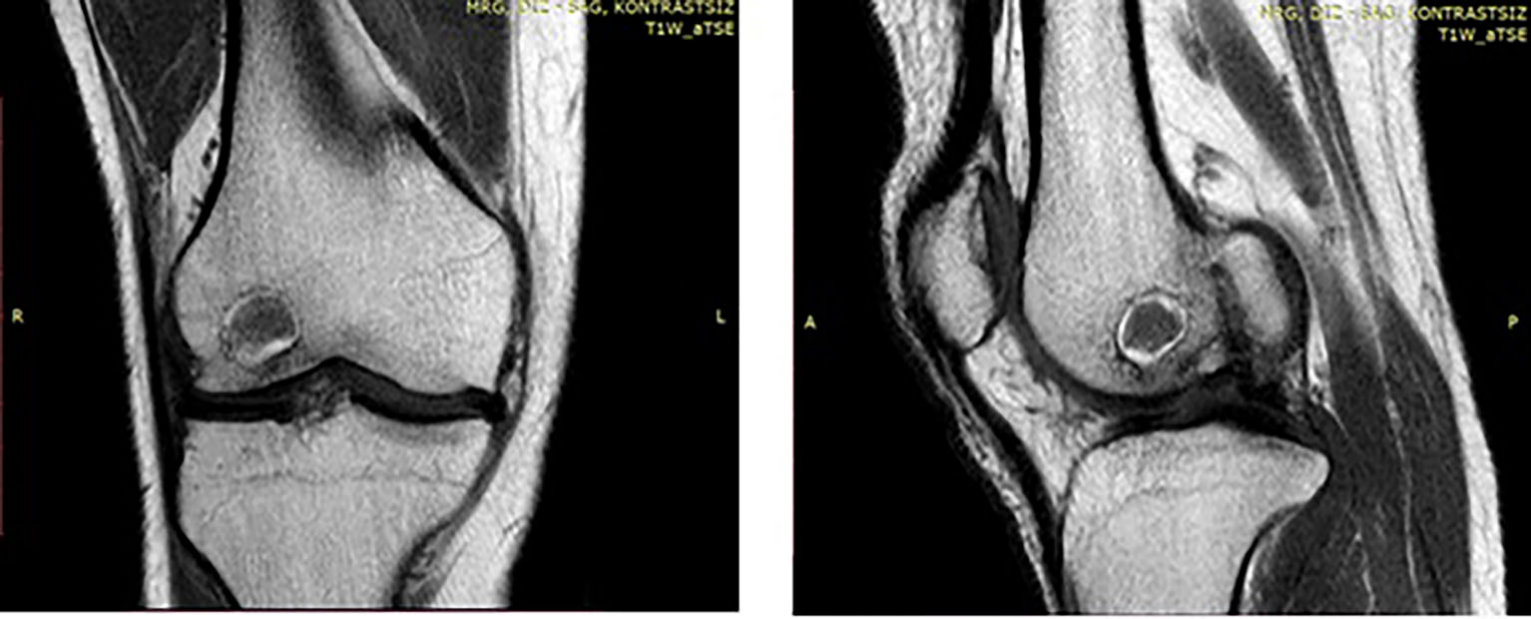
Penumbra sign in the MRI imaging of the patient.

The patient stated that 3 years had passed since the surgery and there has been no history of trauma during this period. It was learned that he had no complaints other than knee pain and limping in the last month, and the pain was only around the right knee area. There was no history of tuberculosis or any other chronic illness. On physical examination, his general condition was fair, conscious, oriented, and cooperative. Range of motion (ROM) in the right knee joint was limited and painful. There was no heat increase or redness around the knee. There was also no discharge or open wound in the knee area. In the subsequent X-ray imaging, two screws were seen in the distal femur and a radiolucent lesion surrounded by a well-defined sclerotic rim anterior to the screws was observed. In laboratory tests, C-reactive protein (CRP) was 14.4 mg/dL (reference range: 0-5 mg/dL), erythrocyte sedimentation rate (ESR) was 18 mm/h (reference range: 0-20 mm/h), and leukocyte count was 9.2×10^3^/mm^3^ (reference range: 4.1-10.4×10^3^/mm^3^). MRI was planned due to the suspicious radiolucent area in the X-ray imaging. Low-density fluid and an oval lesion of approximately 2 cm surrounded by hyperintense granulation tissue in the distal femur metaphysis were observed in the MRI (Figure 3).

### Differential diagnoses

The penumbra sign, which weakly signals hyperintense content with a characteristic border of granulation tissue, as seen in the main abscess content in the MRI imaging (Figure 3), is an early sign of osteomyelitis. It can also be seen in tuberculosis osteitis, osteoid osteoma, chondrosarcoma, eosinophilic granuloma, benign cystic neoplasm, and intraosseous ganglion (13). Tuberculosis osteitis was not considered in the differential diagnosis of our patient due to the lack of a history of tuberculosis. Infectious parameters were considered before bone tumors due to the patient's surgical history three years earlier. If there was no surgical history, benign and malignant bone tumors should have also been included in the differential diagnoses.

### Procedures

**Figure 4:**
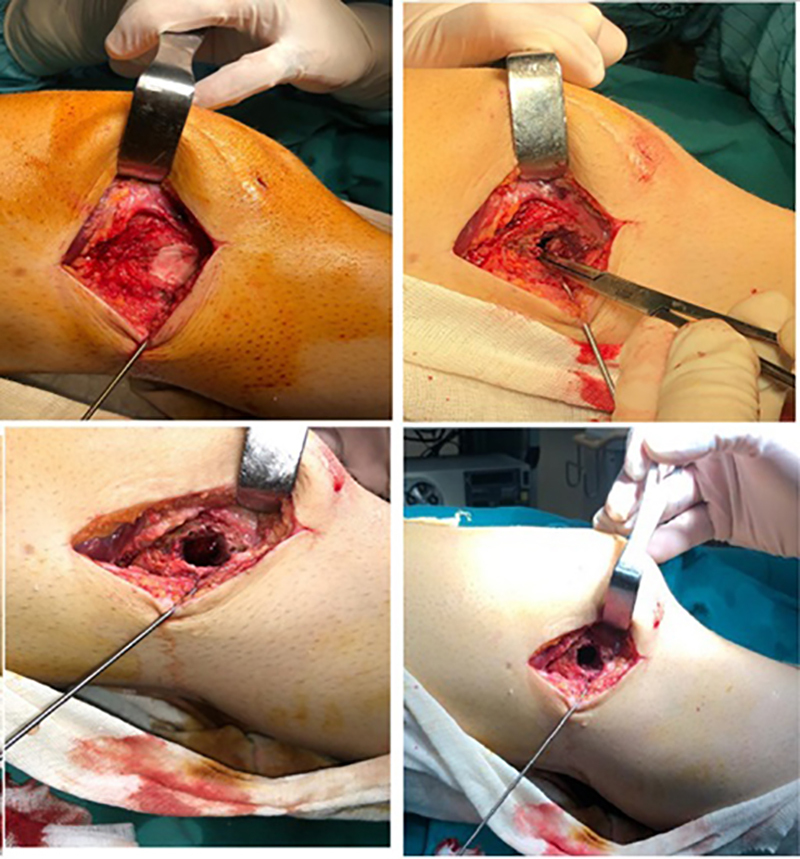
Appearance of the lesion during the surgery.

After the evaluation of the patient, osteomyelitis was considered. Surgical treatment and intraoperative culture were planned. First, arthroscopic intervention was performed on the knee joint, during which it was observed that the screws placed in the patient's knee three years before were not infected and the osteochondral fragment had healed stably (Figure 1). As the screw was intra-articular and the abscess was in the extra-articular area, we thought that removing the screw during surgery could risk transmitting the infection into the joint and causing septic arthritis. For this reason, we did not consider performing this procedure while the abscess was drained. Then, the distal femur was reached through lateral incision and the vastus lateralis muscle was lifted with a pedicle from the posterior, and the lateral femur was reached. A cortical window was opened from the lateral side of the femur under scope control and direct access to the abscess cavity was achieved. Abscess drainage was then performed and the granulation tissue surrounding the lesion was visualized and cleaned with the help of a curette. The cavity created by the abscess (Figure 4), which was soft in texture, was washed with antiseptic and antibiotic solutions (clindamycin phosphate; Klindan^®^ 300 mg, Bilim Pharmaceuticals, Turkey) and appropriate local debridement was performed. The cavity was then thoroughly dried and filled with polymethylmethacrylate (PMMA; Powerbone^®^, Pioneer of Health, Turkey) cement to achieve complete healing and structural support. Antibiotics were not added to the bone-inducing cement to avoid changing cement properties. After the placement of PMMA cement, the surgery was completed.

### Follow-up

During hospitalization, the patient received analgesic (intravenous diclofenac sodium, 50 mg 2×1) and antibiotic (intravenous teicoplanin 400 mg loading dose, 2×400 mg, 3 doses; maintenance therapy 1×400 mg intravenous, for 6 weeks) treatment. Microbiological evaluation of the abscess content identified *Staphylococcus aureus* as the cause of the abscess and that it was sensitive to teicoplanin, so the treatment was continued, and on the 12th day of treatment, the patient's condition improved, and he was discharged. The patient was followed-up for 6 weeks, during which no pathology was observed, and pain complaints significantly decreased in the 3rd week and completely disappeared in the 2nd month.

## Discussion

Clinical suspicion was the first and most important factor for the accurate diagnosis of the case. Although an MRI Image is used to differentiate the disease in Ewing sarcoma and osteomyelitis cases, penumbra sign is more indicative for infection because the transition zones of Ewing sarcoma are more distinct and sharp (14,15). In these cases, early treatment of Brodie's abscess not only speeds up patient recovery but also eliminates complications such as septic arthritis. In addition, filling the defect with bone-inducing cement can correct bone weakness and enable bone reconstruction (16,17).

MRI is the best imaging method for the definitive diagnosis of osteomyelitis due to its ability to show edema in the bone, confirm the abscess, and define extraosseous spread. If MRI is not possible, nuclear medicine imaging and computed tomography (CT) are alternative imaging methods for these cases. The limited sensitivity of CT in showing soft tissue and bone marrow edema is a limiting factor. Therefore, it cannot rule out early osteomyelitis. For this reason, it is an alternative when MRI imaging is not used.

The penumbra sign, which shows weak hyperintense signals depending on the main content of the abscess and is characterized by the edge of the lesion surrounded by granulation tissue, is an early sign of osteomyelitis in MRI. McGuiness et al. (17) reported that the penumbra sign had a high specificity of 96% but a low sensitivity of 27% for musculoskeletal infections. In our case, the penumbra sign detected with MRI was an important early finding for estimating osteomyelitis and accelerated the diagnosis.

In our case, *Staphylococcus aureus* was detected in the microbiological evaluation of the material taken from the abscess, and in the literature, *Staphylococcus aureus* is reported as the most common underlying cause of osteomyelitis (3,8), which supports our case presentation.

Laboratory test results are usually within normal limits in Brodie's abscesses. However, increases in CRP, ESR, and leukocyte counts can be seen sometimes (5). In our case, only CRP was slightly elevated and did not indicate any acute osteomyelitis diagnosis.

Osteomyelitis can present with a slow and relatively benign clinical course and can cause difficulty in determining the treatment due to the need for conservative antibiotic therapy or surgical debridement combined with antibiotics. Many surgeons prefer surgical treatment for lesions that cannot be distinguished from aggressive bone tumors because biopsy is required for aggressive lesions (18,19). In our case, although malignant lesions were not primarily considered, surgical treatment was preferred because of the unclear nature of osteomyelitis and the possibility of debridement.

### Conclusion

In non-traumatic cases presenting with persistent bone pain, osteomyelitis and benign and malignant bone lesions are often confused and challenging for the clinician. MRI is often used in differential diagnosis, and the detection of characteristic imaging signs such as penumbra sign accelerates the diagnosis. In this context, it is useful for emergency department clinicians, who are often the first point of contact for these patients, to be cautious and recognize these signs so that early diagnosis and treatment can be initiated.
